# Electrostatic Surface Potential as a Key Parameter in Virus Transmission and Evolution: How to Manage Future Virus Pandemics in the Post-COVID-19 Era

**DOI:** 10.3390/v15020284

**Published:** 2023-01-19

**Authors:** Jacques Fantini, Fodil Azzaz, Henri Chahinian, Nouara Yahi

**Affiliations:** Department of Biology, Faculty of Medicine, University of Aix-Marseille, INSERM UMR_S 1072, 13015 Marseille, France

**Keywords:** pandemic, vaccine, antiviral, SARS-CoV-2, HIV-1, MERS-CoV, monkeypox virus, influenza virus, lipid raft, ganglioside, neutralization, electrostatic surface potential

## Abstract

Virus-cell interactions involve fundamental parameters that need to be considered in strategies implemented to control viral outbreaks. Among these, the surface electrostatic potential can give valuable information to deal with new epidemics. In this article, we describe the role of this key parameter in the hemagglutination of red blood cells and in the co-evolution of synaptic receptors and neurotransmitters. We then establish the functional link between lipid rafts and the electrostatic potential of viruses, with special emphasis on gangliosides, which are sialic-acid-containing, electronegatively charged plasma membrane components. We describe the common features of ganglioside binding domains, which include a wide variety of structures with little sequence homology but that possess key amino acids controlling ganglioside recognition. We analyze the role of the electrostatic potential in the transmission and intra-individual evolution of HIV-1 infections, including gatekeeper and co-receptor switch mechanisms. We show how to organize the epidemic surveillance of influenza viruses by focusing on mutations affecting the hemagglutinin surface potential. We demonstrate that the electrostatic surface potential, by modulating spike-ganglioside interactions, controls the hemagglutination properties of coronaviruses (SARS-CoV-1, MERS-CoV, and SARS-CoV-2) as well as the structural dynamics of SARS-CoV-2 evolution. We relate the broad-spectrum antiviral activity of repositioned molecules to their ability to disrupt virus-raft interactions, challenging the old concept that an antibiotic or anti-parasitic cannot also be an antiviral. We propose a new concept based on the analysis of the electrostatic surface potential to develop, in real time, therapeutic and vaccine strategies adapted to each new viral epidemic.

## 1. Introduction

Our experience in teaching biochemistry and molecular biology at university level has allowed us, over the years, to identify major concepts in biology that are insufficiently covered in biology courses [1]. Among these concepts we can cite the multiple functions of water molecules in biology [2], the temporal dimension of biological processes [3,4], quantum phenomena at work in biology [5], and the electrostatic surface potential of biomolecules [6]. This latter concept has taken on major importance over the past three years in explaining the structural dynamics of SARS-CoV-2 variants [7]. More generally, the electrostatic surface potential is a key element for understanding the evolution of viruses [8,9] and, more specifically, the evolution of virus-host relationships [10]. It therefore seemed important to us to devote this review article to defining the role of the electrostatic surface potential in the evolution of SARS-CoV-2 and other viruses, and to draw possible solutions from this in the face of future viral pandemics.

## 2. Definition of the Electrostatic Surface Potential

Electrostatic interactions play a central role in biology [11]. Intuitively, molecular interactions can be summed up in a double complementarity: geometric and electrostatic. An electronegative hollow is thus naturally adapted to be occupied by an electropositive protuberance [1]. This concept applies at several levels in biology, from molecular interactions to cell–cell associations, and vice versa to different types of repulsion. Although widely developed in the 20th century, it was not until 1982 with the advances in computer graphics that it was possible to visualize the electrostatic potential of biological macromolecules [6]. A universal color code was then adopted: red for electronegative zones, blue for electropositive zones, and white for neutral zones. In this princeps article, the authors represented for the first time the surface electrostatic potential of not only trypsin and an inhibitor attached to the enzyme, but also a DNA-protein complex. This visualization made obvious the notions of geometric and electrostatic complementarities, which represented a major advance for drug design.

A reflective exercise created for our students in the university’s Evolutionary Biology course will allow us to illustrate the scope of this concept. Consider a series of peptide motifs of a virus protein whose amino acid sequence has gradually evolved over time (from t1 to t6):

t1: AEDEEDLDA

t2: AKDEEDLDA

t3: AKDERDLDA

t4: AKDERDLKA

t5: AKDRRDLKA

t6: AKDRRKLKA

**Figure 1:**
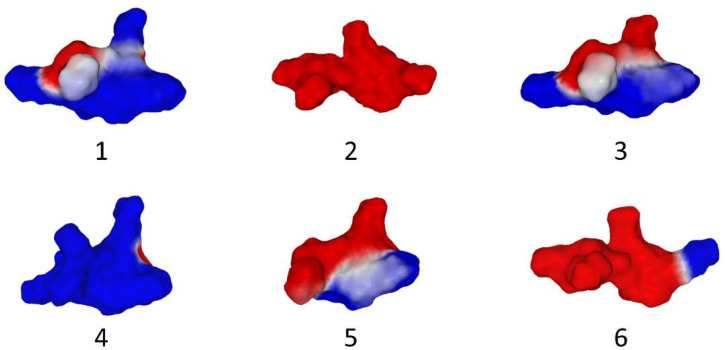
Electrostatic surface potential of an evolving peptide motif (see text for the amino acid sequences). Note that both the electrostatic surface potential and the shape of the motifs are affected by amino acid changes. The purpose of the exercise is to assign each peptide (represented by its electrostatic surface potential and identified by a number) to its corresponding amino acid sequence. Blue, positive; red, negative; white, neutral.

Let us now look at Figure 1 in which the surface electrostatic potential of each of these patterns is represented in a random order. The question is simple: can we attribute to each peptide sequence its corresponding surface electrostatic potential?

The answer is: yes, of course. First you must calculate the net charge of each peptide sequence at pH7: negatively charged side chains (D, aspartic acid; E, glutamic acid) are bold; positively charged side chains (K, lysine; R, arginine) are bold and underscored.

t1: A**EDEED**L**D**A: −6

t2: A**KDEED**L**D**A: −4

t3: A**KDERD**L**D**A: −2

t4: A**KDERD**L**K**A: 0

t5: A**KDRRD**L**K**A: +2

t6: A**KDRRK**L**K**A: +4

The relative position of negatively charged, positively charged, or neutral amino acids will help you assign each sequence its electrostatic surface potential (Figure 1):

t1: A**EDEED**L**D**A: Number 2 (all red)

t2: A**KDEED**L**D**A: Number 6 (mostly red + small lateral blue spot)

t3: A**KDERD**L**D**A: Number 5 (still mostly red, but blue zones become larger)

t4: A**KDERD**L**K**A: Number 3 (50% red, 50% blue)

t5: A**KDRRD**L**K**A: Number 1 (mostly blue + two small red spots)

t6: A**KDRRK**L**K**A: Number 4 (mostly blue + small lateral red spot)

**Figure 2:**
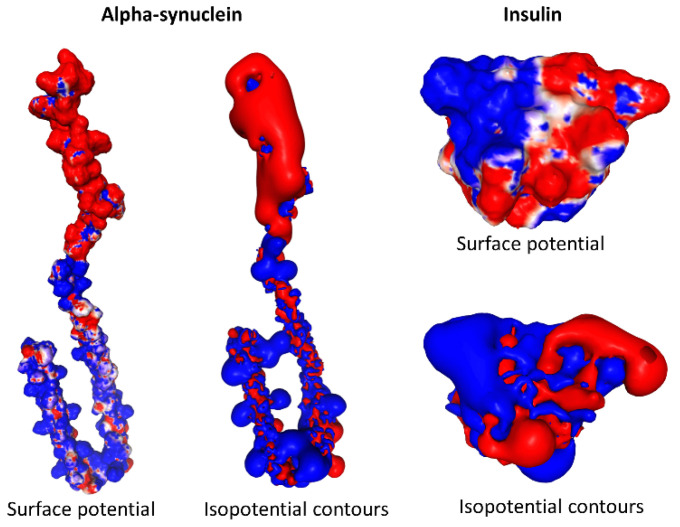
Surface electrostatic potential and isopotential contours. The models (pdb files 1XQ8 and 3I40 for alpha-synuclein and insulin, respectively) were generated by PBEQ-Solver in the biomolecular simulation program CHARMM. Two renditions are shown for each protein (electrostatic surface potential and isopotential contours). Blue, positive; red, negative; white, neutral.

We thus visualized the electrostatic logic of a biomolecule by converting the concept of surface electrostatic potential into a tricolor code. Schematically two types of analysis can visualize the electrostatic potential of a protein: (i) the electrostatic surface potential which superimposes the distribution of charges on the relief of the protein, and (ii) its spatial distribution (isopotential contours). Both types of representations are given in Figure 2. Using isopotential contours is especially useful to highlight slight differences on protein surface, e.g., when studying the evolution of mutants [12]. However, this representation gives a distorted picture of the protein structure, as can be seen in Figure 2. In the next part of this review, we will illustrate the impact of the electrostatic potential concept using different examples from biology.

## 3. Biological Significance of the Electrostatic Surface Potential

Technically, the electrostatic potential can be generated by solving the Poisson–Boltzmann equations, using the partial charges of all the atoms belonging to a given area of a molecule [13,14], or by using Coulomb’s law [15]. In this review, we generally used Molegro Molecular Viewer (http://molexus.io/molegro-molecular-viewer/, accessed on 14 January 2023) to visualize the surface electrostatic potential. The electrostatic potential measured and illustrated by Molegro Molecular Viewer is the sum of the Coulomb potentials for each atom of the considered molecule, with a distance-dependent dielectric constant. Alternatively, the biomolecular simulation program CHARMM (http://www.charmm-gui.org, accessed on 14 January 2023) proposes the PBEQ-Solver module to solve the finite-difference Poisson–Boltzmann equation of submitted proteins. PBEQ-Solver gives the calculated electrostatic potential on the solvent-accessible surface as well as iso-electrostatic potential contours [16,17].

Biomolecules present immense diversity at the level of their surface potential, which is expressed not only by well-delimited zones presenting a positive or negative potential, but also by distribution gradients of charges visualized by the shades of the color code blue-red-white. However, there are major trends among biomolecules. Nucleic acids are negatively charged because of the phosphate groups of the 3′-5′-phosphodiester bonds [18]. Lipid rafts, which are rich in glycosphingolipids [19], are globally electronegative, especially when these glycosphingolipids are gangliosides [20]. Proteins are more ambivalent, as they can have anionic, cationic, and most often both amino acids in infinitely varied proportions. Unlike nucleic acids and lipid rafts, the electrostatic surface potential of proteins is therefore a characteristic property of each protein that needs to be studied independently for each protein. Moreover, as we have seen in Figure 1 with selected peptides, evolution by point mutations can have a very strong impact on the surface potential of a particular region of the protein, with potentially important functional consequences.

If we had to illustrate with a single example the importance of the electrostatic surface potential in biology, we would cite red blood cells. Despite their very large number in the blood, these cells do not aggregate under physiological conditions. On the contrary, two red blood cells repel each other if they get too close. The reason is that the plasma membrane of red blood cells contains negatively charged glycoproteins and glycolipids, which creates a repulsive electric potential (zeta) between cells and prevents their aggregation in the bloodstream [21]. Correspondingly, both neuraminidase (which removes negatively charged sialic acids from glycoproteins and gangliosides) and protease treatments of red blood cells reduce charge surface density and promote agglutination [22,23].

**Figure 3:**
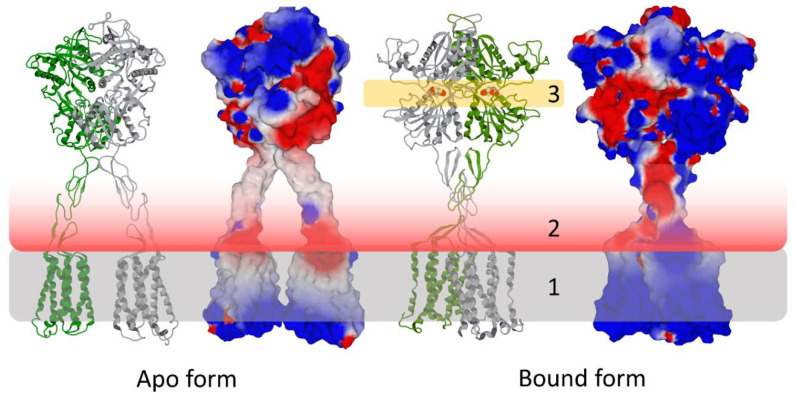
Post-synaptic membrane gangliosides (1, grey zone) generate a repulsive electrostatic field (2, red to white gradient) that determines the position of the agonist binding site (3, orange zone) in the extracellular Venus flytraps of the dimeric metabotropic glutamate receptor 5 (mGluR5). The structure of mGluR5 was retrieved from pdb 6N52.

Another important area of biology in which the electrostatic surface potential plays a major role is the synapse [1]. Post-synaptic membranes are enriched in mono, di-, and tri-sialylated gangliosides [24,25] which confer a strong electronegative field [1,26]. This electrostatic shield repels glutamate away from the neuronal membrane, thus limiting the risk of excitotoxicity [1]. However, this mechanism implies that the binding site of glutamate and its agonists on their receptors is located outside the influence of the negative charges of gangliosides. This is particularly clear on metabotropic receptors, such as mGluR5, whose oversized Venus flytrap domain binds glutamate at a distance of about 80 Å from the membrane (Figure 3) [27]. Thus, we should consider not only the surface electrostatic potential of a protein, but also its dielectric constants which express the influence of the environment on protein–protein and protein–ligand interactions [28].

**Figure 4:**
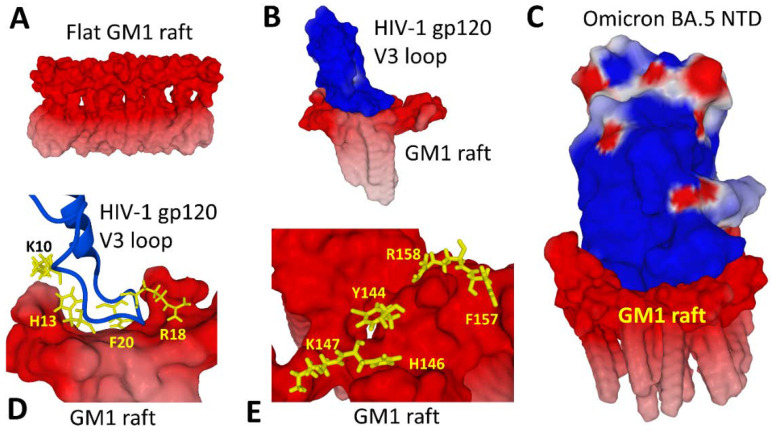
The electrostatic logic of virus-raft interactions. (**A**) The typical flat surface of a GM1 raft before virus binding. (**B**) A typical loop-shaped ganglioside binding domain (HIV-1 gp120 V3 loop). Note the raft curvature induced by the binding reaction. (**C**) Attachment of the N-terminal domain (NTD) of SARS-CoV-2 Omicron BA.5 variant to a GM1 raft. In this case, the membrane curvature induced by the binding reaction is particularly obvious. (**D**) Key amino acid residues controlling the binding of HIV-1 gp120 V3 loop to a GM1 raft. (**E**) Key amino acid residues controlling the binding of the BA.5 NTD to a GM1 raft. The structures were retrieved from pdb 1CE4 (V3 loop) and 7BNM (Omicron spike protein) and modeled with Hyperchem.

In the plasma membrane, gangliosides are not randomly distributed but concentrated in particular microdomains, referred to as lipid rafts [19]. Rafts are a privileged site of attack for many pathogens, especially viruses [29,30,31,32]. There are many explanations for this phenomenon, the first being topological. Rafts are relatively flat areas of the plasma membrane [33]. They therefore represent very accessible landing strips for pathogens (Figure 4A). The second reason is that many virus receptors and/or co-receptors are associated with lipid rafts. By directly targeting the rafts, viruses therefore have facilitated access to these receptors. This is the case for ACE2, the main SARS-CoV-2 receptor, and for CD4, the classical HIV-1 receptor. This situation moreover complicates the very notion of “virus receptor”, since in certain cases raft gangliosides (or raft glycosphingolipids) can fulfill the virus receptor function. Indeed, gangliosides have been identified as bona fide receptors for various viruses, including influenza [34], Sendaï [35], SV40 [36], polyomaviruses [37], and rotavirus [38]. HIV-1 also uses ganglioside GM3 as a fusion cofactor [39,40,41,42] and galactosylceramide (GalCer) as an alternative receptor to infect CD4-negative neural [43] and intestinal epithelial cells [44].

The structural basis of the interaction between gangliosides and these viruses is a ganglioside binding domain [19,26,45], which may be either a small loop [46] (Figure 4B), a large flat surface [47] (Figure 4C), or an annular binding domain [48]. Despite the lack of amino acid sequence homology, these domains display a combination of aromatic and cationic residues which are particularly adapted for optimal ganglioside binding [26,49]. For instance, the binding of HIV-1 gp120 to a cluster of three GM1 molecules involves a central aromatic residue (F20) surrounded by two cationic amino acids (K10, R18) and one histidine (H13) (Figure 4D). The binding of the N-terminal domain (NTD) of SARS-CoV-2 to a GM1 raft involves a similar panel of amino acids (Figure 4E): aromatic (Y144, F157), cationic (K147, R158), and one histidine (H146). A notable feature of this attachment of a virus protein to a raft is the deformation that this molecular interaction causes in the organization of gangliosides, inducing a local curvature of the raft, a phenomenon facilitated by cooperative interactions between gangliosides. This curvature allows the raft to form a kind of stabilization cocoon. The binding reaction is cooperative, starting with one ganglioside molecule and gradually reinforced by its neighbors in the raft. The kinetics of the reaction are controlled by attractive electrostatic forces between the electronegative (red) surface of the raft and the electropositive (blue) surface of the virus protein. Molecular dynamics simulations performed with the Hyperchem software [50] showed that the conformational rearrangements needed to fit the raft surface concerns chiefly the amino acid side chains of the virus protein that interact with gangliosides, rather than the secondary or tertiary structure of the protein. In other words, it is the lipid raft that adapts its shape to the viral protein surface, not the reverse. This phenomenon is well illustrated by comparing the raft surface before (Figure 4A) and after binding to the V3 loop of HIV-1 gp120 (Figure 4B) or to the SARS-CoV-2 Omicron Spike protein (Figure 4D).

## 4. Electrostatic Surface Potential in HIV-1 Evolution

**Figure 5:**
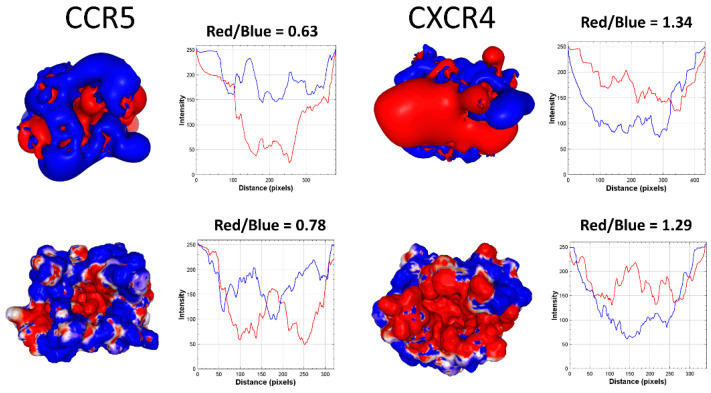
Electrostatic surface potential of CCR5 and CXR4 co-receptors. The structures were modeled from the coordinates of pdb 7F1T (CCR5) and 3OE0 (CXCR4). The upper panels show the isopotential contours and the lower panels the surface electrostatic potential as calculated by PBEQ-Solver. The determination of the blue (electropositive) and red (electronegative) areas performed with the ImageJ software showed that the isopotential contours give a clearer estimate of the relative charge distribution.

The main HIV-1 receptor is the CD4 glycoprotein expressed by certain immune cells [51]. However, one of the most surprising characteristics of this retrovirus is the ability to use, in addition to CD4, a co-receptor necessary for the process of fusion between the virus envelope and the plasma membrane of the host cell [52]. When an individual is infected with HIV-1, it is usually a strain using the CCR5 co-receptor that is transmitted [53]. Then, as the virus evolves in the patient, a co-receptor switch occurs, allowing the virus to use another co-receptor, CXCR4 [54]. In the first case, we speak of R5 viruses; in the second case, of X4 viruses. The transition is marked by viruses that can use both types of co-receptors: these are the R5X4 viruses [55]. The co-receptor switch (from CCR5 to CXCR4) is associated with the emergence of more aggressive viruses, inducing a more rapid decline of CD4^+^ lymphocytes [56,57]. The structural basis of this evolution is largely caused by an increase in the net charge of the V3 loop, due to an accumulation of mutations that dramatically affect its electrostatic surface potential [10]. This mechanism can be explained by considering the electrostatic surface potential of CCR5 and CXCR4 (Figure 5), which must be complementary to the V3 loop.

According to the analysis of Figure 5 performed with the ImageJ software [58], the isopotential contours of CXCR4 are 2.12 times more electronegative than CCR5 (and 1.65 times for the determinations based on the electrostatic surface potential). Correspondingly, the net charge of the V3 loop, and thus its electropositive surface potential, gradually increases as the virus evolves in an infected individual. When it reaches a threshold value (+4 or +5), the virus becomes able to use both CCR5 and CXCR4. Above this value, the virus definitely switches and uses CXCR4 [10]. Thus, the value of the net charge of the V3 loop makes it possible to predict the type of co-receptor used by each HIV-1 isolate. This type of analysis allows an understanding of this retrovirus’ evolution since its emergence in the human species, giving us keys to anticipate its future evolution [59].

**Figure 6:**
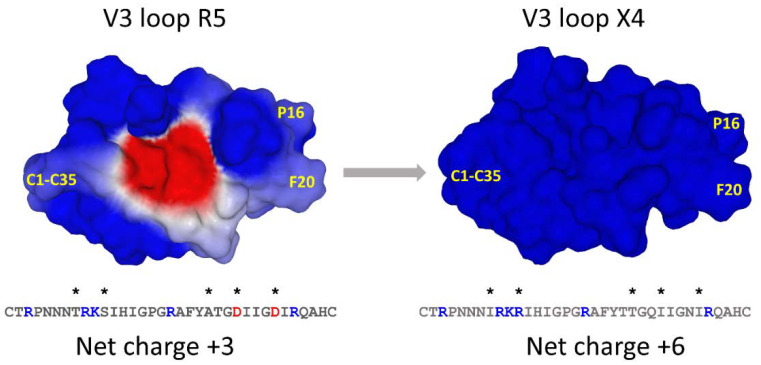
V3 loop evolution associated with the co-receptor switch. Amino acid changes are indicated by an asterisk (*): blue, amino acid with a positive charge; red, amino acid with a negative charge. The position of the disulfide bridge between cysteine C1 and C35 is indicated. The crown of the V3 loop is on the right side of the structure (GPGRAF motif). These structures were modeled from the coordinates of pdb 1CE4.

A representative example of V3 loop sequence evolution associated with the co-receptor switch is given in Figure 6. The V3 loop of a typical R5 isolate has a net positive charge of +3, which results from the compensation of 5 cationic and 2 acidic residues [60]. The electrostatic surface potential of this V3 loop is globally electropositive, but with a large central electronegative spot generated by aspartic acids D25 and D29. The evolution of this V3 loop led to a net charge of +6 due to the presence of a new cationic residue (R11) and the substitution of D25 and D29 by two amide residues (Q25 and N29) which are not electrically charged. These changes are associated with the R5 🡪 X4 co-receptor switch. It is obvious that the electrostatic surface potential of this X4 V3 loop, which is highly electropositive, is well adapted to interact with the large electronegative receptacle of CXCR4 (Figure 5).

Aside from mutations that increase the electrostatic potential, the X4 V3 loop lacks the glycosylation site NNT which is changed to NNI (Figure 6). Glycans display an electronegative potential [61] which favors the use of CCR5 rather than CXCR4 [10,60]. Thus, the lack of a glycosylation site in the X4 V3 loop can be interpreted as the result of the selective pressure that allows the emergence of viruses with an increased electropositive surface potential.

Heterosexual transmission of HIV-1 is an imperfectly known process during which a single virus is selected and transmitted to the recipient. In the majority of cases, even if the sexual secretions contain a mixture of R5, R5X4, and X4 viruses, the transmitted founder virus is a R5 virus [62]. The mechanisms responsible for this selection are still the subject of debate. Several hypotheses have been put forward, some suggesting positive selection, others negative selection. Among the multiple barriers that could protect the recipient from X4 viruses, mucus is probably the more efficient, because it is not selective. Human cervical mucus is made of mucins, which are polyanionic glycoproteins [63]. Since the V3 loop of X4 viruses is more cationic than R5 viruses (Figure 6), this may result in the trapping of X4 strains to mucins, leaving the field open to the less electropositive R5 viruses [64]. In the same way, heparan sulfate proteoglycans that cover mucosal surfaces display an electronegative surface potential able to attract and inactivate the V3 loop of X4 viruses [65]. Sulfatides, which are negatively charged glycosphingolipids expressed by vaginal and intestinal epithelial cells, can also selectively inhibit the sexual transmission of highly cationic X4 viruses [66]. Vaginal epithelial cells are not infected by HIV-1 [67], but the specific sequestration of X4 strains by the genital epithelium could also contribute to the HIV-1 selection process [68]. Finally, the predominant transmission of R5 strains after sexual intercourse may also involve the preferential transmigration of R5 viruses associated with monocytes across the endocervical monolayer [69]. Taken together, these elements suggest that there is not a single “gatekeeper” [70] but rather multiple barriers that gradually select R5 over X4 HIV-1 strains after sexual intercourse [64]. Yet the problem is not easy to solve. Indeed, the selection of R5 viruses after direct intravenous contamination (e.g., transfusion with HIV-1 contaminated blood) suggests that post-mucosal gatekeeping mechanisms are also operative [64]. In this case, the infection of macrophages by R5 viruses might play a role, as these cells are less susceptible to cytotoxic lymphocytes [71]. What is clear is that R5 viruses systematically evolve towards X4 strains by increasing the electrostatic surface potential of the gp120 V3 loop by several mechanisms: (i) increase in the frequency of cationic amino acids, (ii) disappearance of electronegative amino acids, and (iii) suppression of the V3 loop glycosylation site (Figure 6) [12].

Differences in co-receptor usage have also been observed between genetic HIV-1 subtypes with a distinct geographical distribution [72]. Interestingly, López de Victoria et al. (2012) elegantly demonstrated that V3 loop subtypes with similar spatial distribution of electrostatic potential cluster together [12]. Thus, for X4 and R5 viruses, the electrostatic surface potential of the V3 loop is a fundamental property that can be used to characterize and classify HIV-1 subtypes. This notion of a reference threshold value to categorize variants, quasi-species, and/or subtypes of HIV-1 is in fact fairly standard for this retrovirus. Indeed, variations in the genomic sequence of HIV-1 subtypes can also be detected retrospectively in RT and/or protease sequence databases, when the divergence with a reference subtype B virus (HXB2) exceeds the cut-off value determined by the algorithm [73].

## 5. Electrostatic Surface Potential in Influenza Virus Evolution

**Figure 7:**
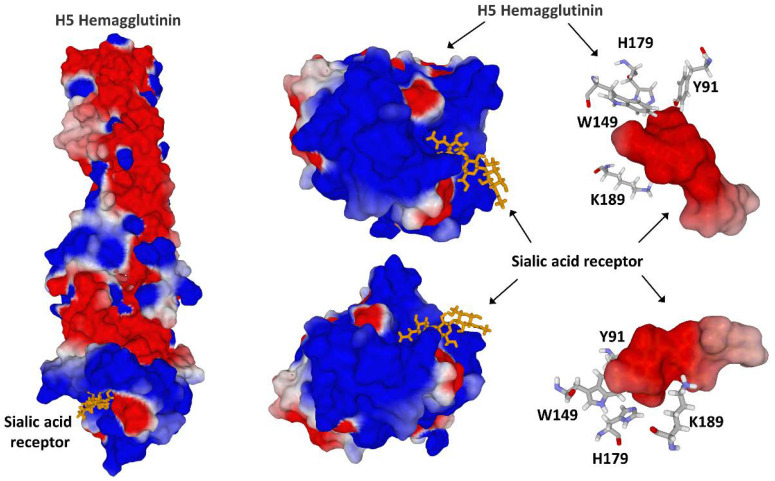
Sialic acid receptor binding site on influenza H5 hemagglutinin (retrieved from pdb 4BGY). The sialic acid receptor binding sites include cationic (K189), aromatic (Y91, W149), and one histidine residue (H179).

If there is a virus for which the electrostatic surface potential should be studied with great interest, it is the influenza virus [9,74,75,76]. The basic reason for this is that this virus uses the sialic acid residues of glycoproteins and gangliosides to infect host cells and spread from animal species to humans, as well as from human to human [77,78,79,80,81]. The sialic acid binding site of influenza virus hemagglutinin displays the same pattern of cationic and aromatic residues as canonic ganglioside binding domains. This is perfectly illustrated by a ferret-transmissible H5 avian influenza virus (Figure 7) [82]. Indeed, the tip of this H5 hemagglutinin displays a high electropositive surface potential that fits with the electronegative potential of the sialic acid receptor. This adaptation renders the virus able to infect several animal species, representing a potential threat for humans. A totally distinct situation has been demonstrated for the bat influenza virus H17N10. In this case, electrostatic potential analyses revealed that its putative receptor-binding site is highly acidic, making it unfavorable to bind any negatively charged sialylated receptors [83]. This study highlights the power of the concept of surface electrostatic potential to predict the spillover of influenza viruses. Any mutation or genetic rearrangement that would render this domain electropositive would be considered a potential signal for future transmission to humans. Focusing on these hot spots would simplify the virological surveillance based on nucleotidic sequence studies. There are some advantages of this strategy. On the one hand, sequence homology is not always related to structural similarity, meaning we may need to consider structural homology instead [46,74]. On the other hand, a classification based on the electrostatic surface potential is immediately informative since it is directly related to virus-host interactions [7]. Furthermore, as developed for the monkeypox virus, there is a consistent overlapping between the cationic ganglioside binding motifs of virus glycoproteins and neutralizing epitopes [48]. In this respect, any increase in the receptor-binding affinity to gangliosides and related sialic acid receptors should alert us [81]. However, this requires complex physicochemical measurements and the real-time availability of recombinant hemagglutinin. Sequencing methods bypass these delicate and time-consuming steps. Identifying and periodically monitoring hot mutational spots in the genomic regions coding for ganglioside binding motifs will give valuable and timely information about the imminence of animal virus outbreaks, possible transmission to humans, and pandemic risks.

## 6. Electrostatic Surface Potential in Coronavirus Evolution

**Figure 8:**
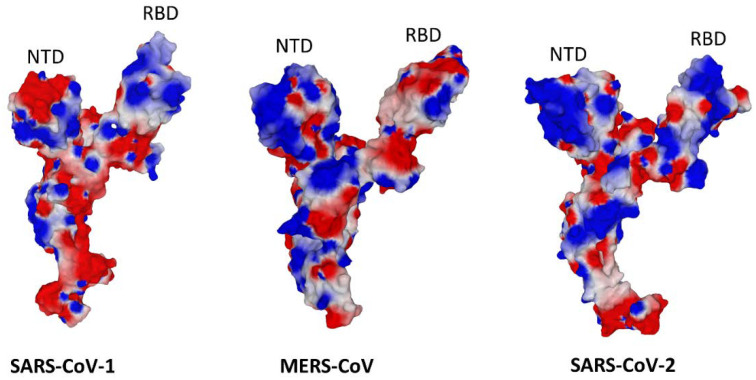
Comparison of the electrostatic surface potential of the three pathogenic coronaviruses for the human species. These structures were retrieved and modeled from pdb 5X5B (SARS-CoV-1), 5X59 (MERS-CoV), and 7BNM (SARS-CoV-2).

Three coronaviruses can trigger severe diseases in infected human individuals: SARS-CoV-1 [84], MERS-CoV [85], and SARS-CoV-2 [86]. The binding of these viruses to the host cell membrane is mediated by a spike protein arranged in a trimer configuration. Each monomer has a typical Y shape where the lateral branches of the letter correspond to the N-terminal domain (NTD) and the receptor binding domain (RBD). Sialic acids, gangliosides, and/or lipid rafts are involved in the entry of these viruses [47,87,88,89,90,91]. In most cases the NTD controls the initial interaction of the virus with lipid raft gangliosides, whereas the RBD is assigned to the recognition of a protein receptor, dipeptidyl peptidase 4 (DPP4, also known as CD26) for MERS-CoV [92], and ACE2 for both SARS-CoV-1 and SARS-CoV-2 [93]. If we compare the NTD of these three coronaviruses, we can see that this domain has evolved from an electronegative protuberance in SARS-CoV-1 to a curved electropositive domain in MERS-CoV, and finally to a flat and mostly electropositive surface for the initial SARS-CoV-2 strain (Figure 8). In parallel, the RBD was significantly rearranged in SARS-CoV-2 to acquire a curved and mostly electropositive surface that fits particularly well with the electronegative surface potential of ACE2 [7]. This evolution ensures both optimized access to lipid rafts through a kinetic effect and a slight increase in the affinity for ACE2, explaining why only SARS-CoV-2 has been pandemic. Moreover, the global electronegative potential of the NTD of SARS-CoV-1 may explain why this virus does not hemagglutinate red blood cells [89], in contrast with MERS-CoV [89] and SARS-CoV-2 [94]. In fact, the ability of a virus to hemagglutinate red blood cells requires the co-expression of a sialic acid recognition motif [95] and an electrostatic surface potential sufficiently positive to abolish the repulsion of these cells due to their zeta potential [94]. MERS-CoV and SARS-CoV-2 fulfill these criteria, whereas SARS-CoV-1 does not.

From the initial strain originating from Asia in 2019, SARS-CoV-2 variants emerged sequentially to rapidly reach a global distribution [96]. In these variants, mutations, deletions, and/or insertions have remodeled the NTD and the RBD according to a double selection pressure: (i) an immune escape progressively decreasing the effectiveness of neutralizing antibodies [97,98,99] and (ii) a faster access to lipid rafts determined by an increase in the electrostatic surface potential of the NTD, which tends to become increasingly electropositive [7]. In parallel, compensation mutations have appeared, allowing the RBD to retain its binding properties to the ACE2 receptor [100,101].

**Figure 9:**
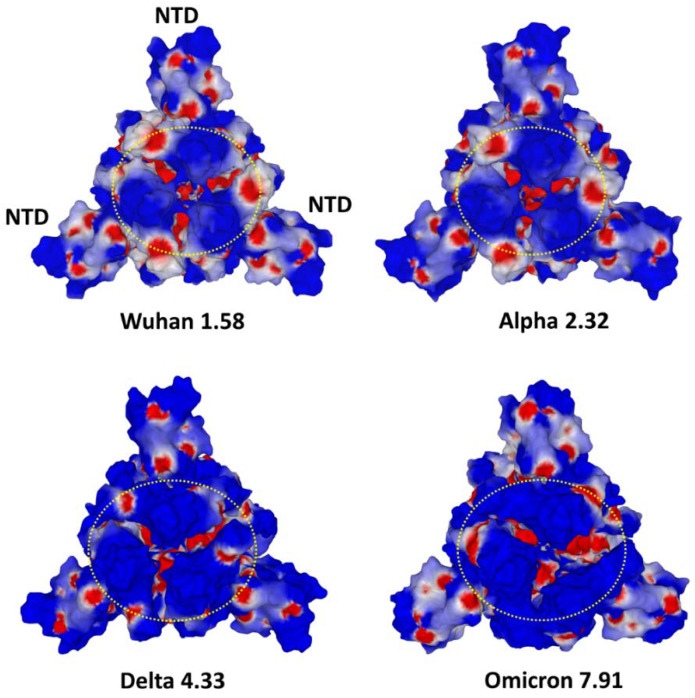
Comparison of the electrostatic surface potential of trimeric spikes of SARS-CoV-2 variants. NTD, N-terminal domain. The three receptor binding domains (RBD) are localized with a yellow dashed circle. The value of the electrostatic surface potential (positive) is indicated for each virus. Note that the NTD of Omicron is less electropositive than that of Delta. However, the surface of Omicron is globally more electropositive than Delta. These structures were retrieved and modeled from pdb file 7BNM.

Analysis of the electrostatic surface potential of the spike trimers shows an overall increase of this potential towards strongly electropositive forms (Figure 9). These global changes mask the differences that may exist in the evolution of a particular domain such as the NTD [102]. Thus, the electrostatic potential of NTD steadily increased in the Alpha to Delta variant series [7]. On the other hand, it has decreased for Omicron, whereas the overall potential of the trimer is markedly increased compared to Delta [94,103], due to the very high electropositivity of the Omicron RBD [102]. Correspondingly, the Omicron spike trimer has a higher hemagglutination capacity compared to other variants, including Delta [94].

This analysis demonstrates the usefulness of considering the surface electrostatic potential as a marker of the evolution of viruses, consistent with the notion that this parameter is one of the essential driving forces of variants [7]. In this respect, a clustering based on the spatial distribution of HIV-1 V3 loop subtypes electrostatic potential was successfully carried out by López de Victoria et al. (2012) [12]. It would also be interesting to compare these analyses with those obtained from antigenic maps of SARS-CoV-2 and influenza virus variants [104,105,106,107]. In this respect, the NTD antigenic mapping revealed a supersite of vulnerability for SARS-CoV-2 [107], which overlaps the ganglioside binding domain [47]. These findings strongly support the concept that ganglioside binding domains coincide with neutralizing epitopes. Thus, identifying these domains in a virus protein is a direct way to develop rapid vaccine formulations.

## 7. Clues for Managing Future Pandemics

How to manage a viral epidemic brutally striking the human species? Very recently we have developed a strategy that could be applied in the event of a new health crisis due to an infectious disease. We have illustrated this strategy using the example of the monkeypox virus [48]. This virus hit the headlines in the summer of 2022 with an unexpected outbreak outside its usual geographical area [108]. Our idea was to identify ganglioside binding motifs in proteins of this virus known to be the target of neutralizing antibodies.

For instance, the ganglioside binding domain located in the NTD of SARS-CoV-2 [47,109] overlaps with the neutralizing epitope of the 4A8 antibody [110].

**Figure 10:**
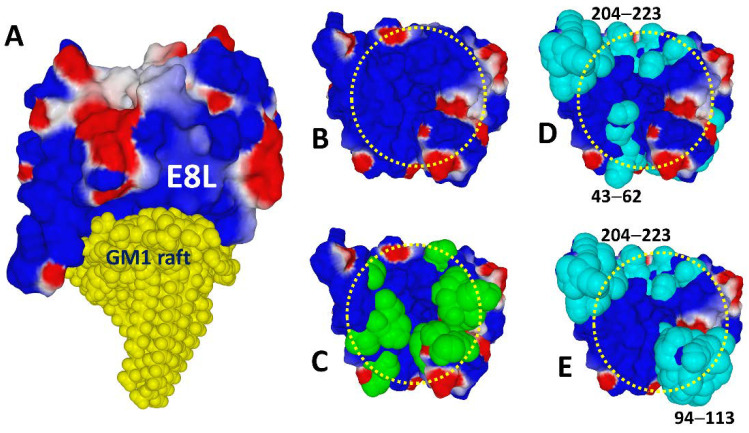
Ganglioside binding domain and B linear epitopes of the Monkeypox virus E8L protein. (**A**) Molecular model of E8L bound to a cluster of gangliosides GM1 in a typical raft organization. (**B**) Electrostatic surface potential of the E8L side facing the GM1 raft. (**C**) Annular distribution of amino acid residues (atomic green spheres) forming the ganglioside binding motif of E8L. (**D**) Epitopes 43–62 and 204–223 (in cyan) of E8L. (**E**) Epitopes 91–113 and 204–223 (in cyan) of E8L. In B–E the annular distribution of amino acids constituting the ganglioside binding domain is indicated by a yellow dashed circle.

A bibliographic search enabled us to identify the cell surface binding protein E8L. We generated a 3D structural model of this protein using data from the UniProt database (https://www.uniprot.org, accessed on 26 December 2022) and the Robetta server (https://robetta.bakerlab.org, accessed on 26 December 2022). By a dedicated molecular modeling approach adapted to the topology of E8L [111], we have determined a possible mode of interaction of this protein, with a cluster of gangliosides mimicking a lipid raft (Figure 10A) [48]. The electrostatic surface potential at the level of the protein domain containing the ganglioside binding motif is strongly electropositive (Figure 10B), in agreement with all the viruses previously studied by our team [26]. This study allowed us to identify a new type of ganglioside binding domain, organized in an annular structure on the surface of the protein (Figure 10C). As expected, this motif contains the usual amino acids necessary for the recognition of gangliosides: cationic (arginine, lysine), aromatic (tyrosine), and histidine amino acids. Thus, apart from the novelty at the level of the annular organization of the motif, the ganglioside binding domain of the monkeypox virus fulfills the molecular criteria governing virus-ganglioside interactions [48].

The second step of our strategy consisted in identifying linear B epitopes [112] that could be easily incorporated into a vaccine formulation in the form of synthetic peptides. Finally, we selected, among all the potential epitopes, those which overlap with the ganglioside binding domain (Figure 10D,E). Given that this analysis is based on the 3D structure of the E8L protein, we were able to determine the most suitable formulation to promote synergies between epitopes and eliminate redundant epitopes. Indeed, neighboring, or even partially superimposed, domains in the 3D structure of the protein may in fact correspond to distant regions in the amino acid sequence. This is the case for epitopes 43–62 and 94–113 of E8L, which show some overlapping (Figure 10). In this case, the best antigenic formula would be to mix synthetic peptides 94–113 and 204–223. These peptides are well conserved among monkeypox virus strains and ideally localized in the structure of the E8L protein to efficiently trigger neutralizing antibodies against monkeypox virus [48].

Until effective vaccines are available, it is possible to use broad-spectrum antivirals to treat infected patients. Here again, the surface electrostatic potential of virus proteins explains the nonspecific antiviral effects of many compounds. Indeed, anionic polymers such as heparan sulfate [113] and glycosaminoglycans [114] are natural antivirals that can bind to the electropositive regions of viruses and prevent their initial adhesion to raft gangliosides. Low molecular weight anionic compounds such as suramin [115] or sulfatide [66], which bind to the V3 loop of HIV-1 gp120, also have potent antiviral activity. Cationic peptide dendrimers which bind to cell surface glycosphingolipids [116] block the infection of not only lymphocytes and macrophages but also CD4-negative cells by several HIV-1 and HIV-2 strains [117,118]. Conversely, synthetic analogues of glycosphingolipids, which interact with the cationic regions of viral proteins, have shown interesting anti-HIV activity [119,120,121,122,123].

More recently we unraveled a new antiviral mechanism for hydroxychloroquine, an antiparasitic drug used by some clinicians for treating SARS-CoV-2 infection [124]. We showed that hydroxychloroquine strongly interacts with raft gangliosides [125] and thus could provide protection against a broad range of viruses that use lipid rafts as the portal of entry [47]. Additionally, our modeling studies identified a potential synergy between hydroxychloroquine and azithromycin, a combination therapy also used for treating SARS-CoV-2 infections [124]. We showed that azithromycin binds to the conserved ganglioside binding domain located in the NTD of SARS-CoV-2 [126], confirming the synergy observed in vitro in infection studies [127]. The antiparasitic drug ivermectin also has broad antiviral properties [128]. In addition, this drug inhibits the hemagglutination of red blood cells induced by the spike trimers of SARS-CoV-2 variants, including Omicron [94].

Despite these important findings, the connection between lipid rafts, surface electrostatic potential, and antiviral activity has not been exploited enough by the medical community. It is a fact that we must now abandon the outdated dogma that an antibiotic cannot cure viral diseases [129]. At the molecular level, such a rigid classification is just nonsense. Azithromycin [130], ivermectin [131], hydroxychloroquine [132], suramin [133], and sulfolipids [134], to mention only a few, whatever they have been used or are used for by clinicians, are also antivirals. Their broad-spectrum antiviral activity has a common target—virus-raft interactions—as these drugs attach themselves either to rafts or to the ganglioside binding domains of viruses. Correspondingly, the antiviral properties of these drugs can be revealed with biological experiments using virus pseudotypes [135]. This assay focuses on viral entry mechanisms, excluding any other step in the replication cycle [136]. Virus pseudotypes have successfully demonstrated the antiviral effect of various antibiotics and antiparasitics, including atovaquone [137], carrimycin [138], azithromycin [139], hydroxychloroquine [140], and suramin [141], as well as glycodendrimers [142] and anionic drugs such as glycosphingolipid sulfatide [66]. By preventing the attachment of viruses to lipid rafts, these compounds could somehow mimic the selection barrier controlling transmission by the R5 strains of HIV-1, to the detriment of the X4 strains, which are blocked by various electronegative structures. The antiviral effect of all these molecules takes its logic from the natural history of virus-cell interactions, which are under the control of the surface electrostatic potential. It is time to incorporate this concept into our therapeutic arsenal in order to reposition old molecules [143,144,145,146] and/or for the design of new antivirals targeting virus-raft interactions [47].

## Data Availability

Not applicable.

## References

[1] Fantini J., Yahi N. (2015). Brain Lipids in Synaptic Function and Neurological Disease: Clues to Innovative Therapeutic Strategies for Brain Disorders.

[2] Chaplin M. (2006). Do we underestimate the importance of water in cell biology?. Nat. Rev. Mol. Cell Biol..

[3] Huang S., Wikswo J. (2006). Dimensions of systems biology. Rev. Physiol. Biochem. Pharmacol..

[4] Brodsky W.Y. (1975). Protein synthesis rhythm. J. Theor. Biol..

[5] McFadden J., Al-Khalili J. (2018). The origins of quantum biology. Proc. R. Soc. A.

[6] Weiner P.K., Langridge R., Blaney J.M., Schaefer R., Kollman P.A. (1982). Electrostatic potential molecular surfaces. Proc. Natl. Acad. Sci. USA.

[7] Fantini J., Yahi N., Azzaz F., Chahinian H. (2021). Structural dynamics of SARS-CoV-2 variants: A health monitoring strategy for anticipating Covid-19 outbreaks. J. Infect..

[8] Repits J., Sterjovski J., Badia-Martinez D., Mild M., Gray L., Churchill M.J., Purcell D.F., Karlsson A., Albert J., Fenyö E.M. (2008). Primary HIV-1 R5 isolates from end-stage disease display enhanced viral fitness in parallel with increased gp120 net charge. Virology.

[9] Heidari A., Righetto I., Filippini F. (2018). Electrostatic variation of haemagglutinin as a hallmark of the evolution of avian influenza viruses. Sci. Rep..

[10] Kalinina O.V., Pfeifer N., Lengauer T. (2013). Modelling binding between CCR5 and CXCR4 receptors and their ligands suggests the surface electrostatic potential of the co-receptor to be a key player in the HIV-1 tropism. Retrovirology.

[11] Honig B., Nicholls A. (1995). Classical electrostatics in biology and chemistry. Science.

[12] López de Victoria A., Kieslich C.A., Rizos A.K., Krambovitis E., Morikis D. (2012). Clustering of HIV-1 subtypes based on gp120 V3 loop electrostatic properties. BMC Biophys..

[13] Fogolari F., Brigo A., Molinari H. (2002). The Poisson–Boltzmann equation for biomolecular electrostatics: A tool for structural biology. J. Mol. Recognit..

[14] Sharp K.A., Honig B. (1990). Calculating total electrostatic energies with the nonlinear Poisson-Boltzmann equation. J. Phys. Chem..

[15] Murray J.S., Politzer P. (2011). The electrostatic potential: An overview. Wiley Interdiscip. Rev. Comput. Mol. Sci..

[16] Jo S., Vargyas M., Vasko-Szedlar J., Roux B., Im W. (2008). PBEQ-Solver for online visualization of electrostatic potential of biomolecules. Nucleic Acids Res..

[17] Im W., Beglov D., Roux B. (1998). Continuum solvation model: Computation of electrostatic forces from numerical solutions to the Poisson-Boltzmann equation. Comput. Phys. Commun..

[18] Auffinger P., Hashem Y. (2007). Nucleic acid solvation: From outside to insight. Curr. Opin. Struct. Biol..

[19] Fantini J., Garmy N., Mahfoud R., Yahi N. (2002). Lipid rafts: Structure, function and role in HIV, Alzheimer’s and prion diseases. Expert Rev. Mol. Med..

[20] Fantini J., Chahinian H., Yahi N. (2020). Progress toward Alzheimer’s disease treatment: Leveraging the Achilles’ heel of Aβ oligomers?. Protein Sci. A Publ. Protein Soc..

[21] Fontes A., Fernandes H., Barjas-Castro M., de Thomaz A., de Ysasa Pozzo L., Barbosa L., Cesar C. (2006). Red Blood Cell Membrane Viscoelasticity, Agglutination, and Zeta Potential Measurements with Double Optical Tweezers.

[22] Luner S.J., Sturgeon P., Szklarek D., McQuiston D.T. (1975). Effects of Proteases and Neuraminidase on RBC Surface Charge and Agglutination: A Klinetic Study 1. Vox Sang..

[23] Van Oss C., Absolom D. (1984). Hemagglutination and the closest distance of approach of normal, neuraminidase-and papain-treated erythrocytes. Vox Sang..

[24] Schnaar R.L. (2016). Gangliosides of the Vertebrate Nervous System. J. Mol. Biol..

[25] Schnaar R.L., Gerardy-Schahn R., Hildebrandt H. (2014). Sialic acids in the brain: Gangliosides and polysialic acid in nervous system development, stability, disease, and regeneration. Physiol. Rev..

[26] Azzaz F., Yahi N., Di Scala C., Chahinian H., Fantini J. (2022). Ganglioside binding domains in proteins: Physiological and pathological mechanisms. Adv. Protein Chem. Struct. Biol..

[27] Koehl A., Hu H., Feng D., Sun B., Zhang Y., Robertson M.J., Chu M., Kobilka T.S., Laeremans T., Steyaert J. (2019). Structural insights into the activation of metabotropic glutamate receptors. Nature.

[28] Amin M., Küpper J. (2020). Variations in proteins dielectric constants. ChemistryOpen.

[29] Mañes S., del Real G., Lacalle R.A., Lucas P., Gómez-Moutón C., Sánchez-Palomino S., Delgado R., Alcamí J., Mira E., Martínez A.C. (2000). Membrane raft microdomains mediate lateral assemblies required for HIV-1 infection. EMBO Rep.

[30] Mañes S., del Real G., Martínez A.C. (2003). Pathogens: Raft hijackers. Nat. Rev. Immunol..

[31] Ripa I., Andreu S., López-Guerrero J.A., Bello-Morales R. (2021). Membrane rafts: Portals for viral entry. Front. Microbiol..

[32] Omasta B., Tomaskova J. (2022). Cellular Lipids—Hijacked Victims of Viruses. Viruses.

[33] Pike L.J. (2003). Lipid rafts: Bringing order to chaos. J. Lipid Res..

[34] Suzuki Y. (1994). Gangliosides as influenza virus receptors. Variation of influenza viruses and their recognition of the receptor sialo-sugar chains. Prog. Lipid Res. 1994, 33, 429–457. Prog. Lipid Res..

[35] Markwell M., Svennerholm L., Paulson J.C. (1981). Specific gangliosides function as host cell receptors for Sendai virus. Proc. Natl. Acad. Sci. USA.

[36] Campanero-Rhodes M.A., Smith A., Chai W., Sonnino S., Mauri L., Childs R.A., Zhang Y., Ewers H., Helenius A., Imberty A. (2007). N-glycolyl GM1 ganglioside as a receptor for simian virus 40. J. Virol..

[37] Maginnis M.S. (2018). Virus–receptor interactions: The key to cellular invasion. J. Mol. Biol..

[38] Rolsma M.D., Kuhlenschmidt T.B., Gelberg H.B., Kuhlenschmidt M.S. (1998). Structure and function of a ganglioside receptor for porcine rotavirus. J. Virol..

[39] Hammache D., Piéroni G., Yahi N., Delézay O., Koch N., Lafont H., Tamalet C., Fantini J. (1998). Specific interaction of HIV-1 and HIV-2 surface envelope glycoproteins with monolayers of galactosylceramide and ganglioside GM3. J. Biol. Chem..

[40] Hammache D., Yahi N., Maresca M., Piéroni G., Fantini J. (1999). Human erythrocyte glycosphingolipids as alternative cofactors for human immunodeficiency virus type 1 (HIV-1) entry: Evidence for CD4-induced interactions between HIV-1 gp120 and reconstituted membrane microdomains of glycosphingolipids (Gb3 and GM3). J. Virol..

[41] Hammache D., Yahi N., Piéroni G., Ariasi F., Tamalet C., Fantini J. (1998). Sequential interaction of CD4 and HIV-1 gp120 with a reconstituted membrane patch of ganglioside GM3: Implications for the role of glycolipids as potential HIV-1 fusion cofactors. Biochem. Biophys. Res. Commun..

[42] Hug P., Lin H.M., Korte T., Xiao X., Dimitrov D.S., Wang J.M., Puri A., Blumenthal R. (2000). Glycosphingolipids promote entry of a broad range of human immunodeficiency virus type 1 isolates into cell lines expressing CD4, CXCR4, and/or CCR5. J. Virol..

[43] Harouse J.M., Bhat S., Spitalnik S.L., Laughlin M., Stefano K., Silberberg D.H., Gonzalez-Scarano F. (1991). Inhibition of entry of HIV-1 in neural cell lines by antibodies against galactosyl ceramide. Science.

[44] Yahi N., Baghdiguian S., Moreau H., Fantini J. (1992). Galactosyl ceramide (or a closely related molecule) is the receptor for human immunodeficiency virus type 1 on human colon epithelial HT29 cells. J. Virol..

[45] Fantini J., Garmy N., Yahi N. (2006). Prediction of glycolipid-binding domains from the amino acid sequence of lipid raft-associated proteins: Application to HpaA, a protein involved in the adhesion of Helicobacter pylori to gastrointestinal cells. Biochemistry.

[46] Mahfoud R., Garmy N., Maresca M., Yahi N., Puigserver A., Fantini J. (2002). Identification of a common sphingolipid-binding domain in Alzheimer, prion, and HIV-1 proteins. J. Biol. Chem..

[47] Fantini J., Chahinian H., Yahi N. (2021). Leveraging coronavirus binding to gangliosides for innovative vaccine and therapeutic strategies against COVID-19. Biochem. Biophys. Res. Commun..

[48] Fantini J., Chahinian H., Yahi N. (2022). A Vaccine Strategy Based on the Identification of an Annular Ganglioside Binding Motif in Monkeypox Virus Protein E8L. Viruses.

[49] Fantini J. (2003). How sphingolipids bind and shape proteins: Molecular basis of lipid-protein interactions in lipid shells, rafts and related biomembrane domains. Cell. Mol. Life Sci. CMLS.

[50] Froimowitz M. (1993). HyperChem: A software package for computational chemistry and molecular modeling. BioTechniques.

[51] Bour S., Geleziunas R., Wainberg M.A. (1995). The human immunodeficiency virus type 1 (HIV-1) CD4 receptor and its central role in promotion of HIV-1 infection. Microbiol. Rev..

[52] Moore J.P., Trkola A., Dragic T. (1997). Co-receptors for HIV-1 entry. Curr. Opin. Immunol..

[53] Moore J.P., Kitchen S.G., Pugach P., Zack J.A. (2004). The CCR5 and CXCR4 coreceptors—Central to understanding the transmission and pathogenesis of human immunodeficiency virus type 1 infection. AIDS Res. Hum. Retrovir..

[54] Regoes R.R., Bonhoeffer S. (2005). The HIV coreceptor switch: A population dynamical perspective. Trends Microbiol..

[55] Briggs D.R., Tuttle D.L., Sleasman J.W., Goodenow M.M. (2000). Envelope V3 amino acid sequence predicts HIV-1 phenotype (co-receptor usage and tropism for macrophages). AIDS.

[56] Vicenzi E., Liò P., Poli G. (2013). The puzzling role of CXCR4 in human immunodeficiency virus infection. Theranostics.

[57] Philpott S.M. (2003). HIV-1 coreceptor usage, transmission, and disease progression. Curr. HIV Res..

[58] Schindelin J., Rueden C.T., Hiner M.C., Eliceiri K.W. (2015). The ImageJ ecosystem: An open platform for biomedical image analysis. Mol. Reprod. Dev..

[59] Tebit D.M., Arts E.J. (2011). Tracking a century of global expansion and evolution of HIV to drive understanding and to combat disease. Lancet Infect. Dis..

[60] Pollakis G., Kang S., Kliphuis A., Chalaby M.I., Goudsmit J., Paxton W.A. (2001). N-linked glycosylation of the HIV type-1 gp120 envelope glycoprotein as a major determinant of CCR5 and CXCR4 coreceptor utilization. J. Biol. Chem..

[61] Jo S., Song K.C., Desaire H., MacKerell A.D., Im W. (2011). Glycan Reader: Automated sugar identification and simulation preparation for carbohydrates and glycoproteins. J. Comput. Chem..

[62] Nijmeijer B.M., Geijtenbeek T.B. (2019). Negative and positive selection pressure during sexual transmission of transmitted founder HIV-1. Front. Immunol..

[63] Wolf D.P., Sokoloski J.E., Litt M. (1980). Composition and function of human cervical mucus. Biochim. Et Biophys. Acta (BBA)-Gen. Subj..

[64] Grivel J.-C., Shattock R.J., Margolis L.B. (2011). Selective transmission of R5 HIV-1 variants: Where is the gatekeeper?. J. Transl. Med..

[65] Moulard M., Lortat-Jacob H., Mondor I., Roca G., Wyatt R., Sodroski J., Zhao L., Olson W., Kwong P.D., Sattentau Q.J. (2000). Selective interactions of polyanions with basic surfaces on human immunodeficiency virus type 1 gp120. J. Virol..

[66] Fantini J., Hammache D., Delézay O., Piéroni G., Tamalet C., Yahi N. (1998). Sulfatide inhibits HIV-1 entry into CD4−/CXCR4+ cells. Virology.

[67] Fantini J., Yahi N., Tourres C., Delezay O., Tamalet C. (1997). HIV-1 transmission across the vaginal epithelium. AIDS.

[68] Berlier W., Bourlet T., Lawrence P., Hamzeh H., Lambert C., Genin C., Verrier B., Dieu-Nosjean M.C., Pozzetto B., Delézay O. (2005). Selective sequestration of X4 isolates by human genital epithelial cells: Implication for virus tropism selection process during sexual transmission of HIV. J. Med. Virol..

[69] Lawrence P., Portran D., Terrasse R., Palle S., Olivier T., Fantini J., Bourlet T., Pozzetto B., Delezay O. (2012). Selective transmigration of monocyte-associated HIV-1 across a human cervical monolayer and its modulation by seminal plasma. AIDS.

[70] Margolis L., Shattock R. (2006). Selective transmission of CCR5-utilizing HIV-1: The’gatekeeper’problem resolved?. Nat. Rev. Microbiol..

[71] Schutten M., Van Baalen C., Guillon C., Huisman R., Boers P., Sintnicolaas K., Gruters R., Osterhaus A.D. (2001). Macrophage tropism of human immunodeficiency virus type 1 facilitates in vivo escape from cytotoxic T-lymphocyte pressure. J. Virol..

[72] Tscherning C., Alaeus A., Fredriksson R., Björndal Å., Deng H., Littman D.R., Fenyö E.M., Albert J. (1998). Differences in chemokine coreceptor usage between genetic subtypes of HIV-1. Virology.

[73] Yahi N., Fantini J., Tourres C., Tivoli N., Koch N., Tamalet C. (2001). Use of drug resistance sequence data for the systematic detection of non-B human immunodeficiency virus type 1 (HIV-1) subtypes: How to create a sentinel site for monitoring the genetic diversity of HIV-1 at a country scale. J. Infect. Dis..

[74] Righetto I., Milani A., Cattoli G., Filippini F. (2014). Comparative structural analysis of haemagglutinin proteins from type A influenza viruses: Conserved and variable features. BMC Bioinform..

[75] Righetto I., Filippini F. (2020). Normal modes analysis and surface electrostatics of haemagglutinin proteins as fingerprints for high pathogenic type A influenza viruses. BMC Bioinform..

[76] Jimenez-Alberto A., Alvarado-Facundo E., Ribas-Aparicio R.M., Castelán-Vega J.A. (2013). Analysis of adaptation mutants in the hemagglutinin of the influenza A (H1N1) pdm09 virus. PLoS ONE.

[77] Weis W., Brown J., Cusack S., Paulson J., Skehel J., Wiley D. (1988). Structure of the influenza virus haemagglutinin complexed with its receptor, sialic acid. Nature.

[78] Kumlin U., Olofsson S., Dimock K., Arnberg N. (2008). Sialic acid tissue distribution and influenza virus tropism. Influenza Other Respir. Viruses.

[79] Franca M., Stallknecht D., Howerth E. (2013). Expression and distribution of sialic acid influenza virus receptors in wild birds. Avian Pathol..

[80] Qi L., Kash J.C., Dugan V.G., Wang R., Jin G., Cunningham R.E., Taubenberger J.K. (2009). Role of sialic acid binding specificity of the 1918 influenza virus hemagglutinin protein in virulence and pathogenesis for mice. J. Virol..

[81] Ayora-Talavera G. (2018). Sialic acid receptors: Focus on their role in influenza infection. J. Recept. Ligand Channel Res..

[82] Xiong X., Coombs P.J., Martin S.R., Liu J., Xiao H., McCauley J.W., Locher K., Walker P.A., Collins P.J., Kawaoka Y. (2013). Receptor binding by a ferret-transmissible H5 avian influenza virus. Nature.

[83] Zhu X., Yu W., McBride R., Li Y., Chen L.M., Donis R.O., Tong S., Paulson J.C., Wilson I.A. (2013). Hemagglutinin homologue from H17N10 bat influenza virus exhibits divergent receptor-binding and pH-dependent fusion activities. Proc. Natl. Acad. Sci. USA.

[84] Weinstein R.A. (2004). Planning for epidemics--the lessons of SARS. N. Engl. J. Med..

[85] Zaki A.M., van Boheemen S., Bestebroer T.M., Osterhaus A.D., Fouchier R.A. (2012). Isolation of a novel coronavirus from a man with pneumonia in Saudi Arabia. N. Engl. J. Med..

[86] Zhou P., Yang X.L., Wang X.G., Hu B., Zhang L., Zhang W., Si H.R., Zhu Y., Li B., Huang C.L. (2020). A pneumonia outbreak associated with a new coronavirus of probable bat origin. Nature.

[87] Lu Y., Liu D.X., Tam J.P. (2008). Lipid rafts are involved in SARS-CoV entry into Vero E6 cells. Biochem. Biophys. Res. Commun..

[88] Li G.-M., Li Y.-G., Yamate M., Li S.-M., Ikuta K. (2007). Lipid rafts play an important role in the early stage of severe acute respiratory syndrome-coronavirus life cycle. Microbes Infect..

[89] Li W., Hulswit R.J., Widjaja I., Raj V.S., McBride R., Peng W., Widagdo W., Tortorici M.A., Van Dieren B., Lang Y. (2017). Identification of sialic acid-binding function for the Middle East respiratory syndrome coronavirus spike glycoprotein. Proc. Natl. Acad. Sci. USA.

[90] Sun X.-L. (2021). The role of cell surface sialic acids for SARS-CoV-2 infection. Glycobiology.

[91] Pirone L., Del Gatto A., Di Gaetano S., Saviano M., Capasso D., Zaccaro L., Pedone E. (2020). A multi-targeting approach to fight SARS-CoV-2 attachment. Front. Mol. Biosci..

[92] Raj V.S., Mou H., Smits S.L., Dekkers D.H., Müller M.A., Dijkman R., Muth D., Demmers J.A., Zaki A., Fouchier R.A. (2013). Dipeptidyl peptidase 4 is a functional receptor for the emerging human coronavirus-EMC. Nature.

[93] Hatmal M.M.M., Alshaer W., Al-Hatamleh M.A., Hatmal M., Smadi O., Taha M.O., Oweida A.J., Boer J.C., Mohamud R., Plebanski M. (2020). Comprehensive structural and molecular comparison of spike proteins of SARS-CoV-2, SARS-CoV and MERS-CoV, and their interactions with ACE2. Cells.

[94] Boschi C., Scheim D.E., Bancod A., Militello M., Bideau M.L., Colson P., Fantini J., Scola B.L. (2022). SARS-CoV-2 Spike Protein Induces Hemagglutination: Implications for COVID-19 Morbidities and Therapeutics and for Vaccine Adverse Effects. Int. J. Mol. Sci..

[95] Stencel-Baerenwald J.E., Reiss K., Reiter D.M., Stehle T., Dermody T.S. (2014). The sweet spot: Defining virus–sialic acid interactions. Nat. Rev. Microbiol..

[96] Guérin P., Yahi N., Azzaz F., Chahinian H., Sabatier J.M., Fantini J. (2022). Structural Dynamics of the SARS-CoV-2 Spike Protein: A 2-Year Retrospective Analysis of SARS-CoV-2 Variants (from Alpha to Omicron) Reveals an Early Divergence between Conserved and Variable Epitopes. Molecules.

[97] Harvey W.T., Carabelli A.M., Jackson B., Gupta R.K., Thomson E.C., Harrison E.M., Ludden C., Reeve R., Rambaut A., Peacock S.J. (2021). SARS-CoV-2 variants, spike mutations and immune escape. Nat. Rev. Microbiol..

[98] Lazarevic I., Pravica V., Miljanovic D., Cupic M. (2021). Immune evasion of SARS-CoV-2 emerging variants: What have we learnt so far?. Viruses.

[99] Hu J., Peng P., Cao X., Wu K., Chen J., Wang K., Tang N., Huang A.-l. (2022). Increased immune escape of the new SARS-CoV-2 variant of concern Omicron. Cell. Mol. Immunol..

[100] Barton M.I., MacGowan S.A., Kutuzov M.A., Dushek O., Barton G.J., Van Der Merwe P.A. (2021). Effects of common mutations in the SARS-CoV-2 Spike RBD and its ligand, the human ACE2 receptor on binding affinity and kinetics. elife.

[101] Moulana A., Dupic T., Phillips A.M., Chang J., Nieves S., Roffler A.A., Greaney A.J., Starr T.N., Bloom J.D., Desai M.M. (2022). Compensatory epistasis maintains ACE2 affinity in SARS-CoV-2 Omicron BA.1. Nat. Commun..

[102] Fantini J., Yahi N., Colson P., Chahinian H., La Scola B., Raoult D. (2022). The puzzling mutational landscape of the SARS-2-variant Omicron. J. Med. Virol..

[103] Pascarella S., Ciccozzi M., Bianchi M., Benvenuto D., Cauda R., Cassone A. (2021). The electrostatic potential of the Omicron variant spike is higher than in Delta and Delta-plus variants: A hint to higher transmissibility?. J. Med. Virol..

[104] Mykytyn A.Z., Rissmann M., Kok A., Rosu M.E., Schipper D., Breugem T.I., van den Doel P.B., Chandler F., Bestebroer T., de Wit M. (2022). Antigenic cartography of SARS-CoV-2 reveals that Omicron BA.1 and BA.2 are antigenically distinct. Sci. Immunol..

[105] Smith D.J., Lapedes A.S., De Jong J.C., Bestebroer T.M., Rimmelzwaan G.F., Osterhaus A.D., Fouchier R.A. (2004). Mapping the antigenic and genetic evolution of influenza virus. Science.

[106] Liu M., Zhao X., Hua S., Du X., Peng Y., Li X., Lan Y., Wang D., Wu A., Shu Y. (2015). Antigenic patterns and evolution of the human influenza A (H1N1) virus. Sci. Rep..

[107] McCallum M., De Marco A., Lempp F.A., Tortorici M.A., Pinto D., Walls A.C., Beltramello M., Chen A., Liu Z., Zatta F. (2021). N-terminal domain antigenic mapping reveals a site of vulnerability for SARS-CoV-2. Cell.

[108] Focosi D., Novazzi F., Baj A., Maggi F. (2022). Monkeypox: An international epidemic. Rev. Med. Virol..

[109] Das T., Mukhopadhyay C. (2022). Identification of possible binding modes of SARS-CoV-2 spike N-terminal domain for ganglioside GM1. Chem. Phys. Lett..

[110] Liu L., Wang P., Nair M.S., Yu J., Rapp M., Wang Q., Luo Y., Chan J.F.W., Sahi V., Figueroa A. (2020). Potent neutralizing antibodies against multiple epitopes on SARS-CoV-2 spike. Nature.

[111] Azzaz F., Yahi N., Chahinian H., Fantini J. (2022). The Epigenetic Dimension of Protein Structure Is an Intrinsic Weakness of the AlphaFold Program. Biomolecules.

[112] Shantier S.W., Mustafa M.I., Abdelmoneim A.H., Fadl H.A., Elbager S.G., Makhawi A.M. (2022). Novel multi epitope-based vaccine against monkeypox virus: Vaccinomic approach. Sci. Rep..

[113] Baba M., Snoeck R., Pauwels R., De Clercq E. (1988). Sulfated polysaccharides are potent and selective inhibitors of various enveloped viruses, including herpes simplex virus, cytomegalovirus, vesicular stomatitis virus, and human immunodeficiency virus. Antimicrob. Agents Chemother..

[114] Lin L.-T., Chen T.-Y., Lin S.-C., Chung C.-Y., Lin T.-C., Wang G.-H., Anderson R., Lin C.-C., Richardson C.D. (2013). Broad-spectrum antiviral activity of chebulagic acid and punicalagin against viruses that use glycosaminoglycans for entry. BMC Microbiol..

[115] Yahi N., Sabatier J.M., Nickel P., Mabrouk K., Gonzalez-Scarano F., Fantini J. (1994). Suramin inhibits binding of the V3 region of HIV-1 envelope glycoprotein gp120 to galactosylceramide, the receptor for HIV-1 gp120 on human colon epithelial cells. J. Biol. Chem..

[116] Delézay O., Hammache D., Fantini J., Yahi N. (1996). SPC3, a V3 loop-derived synthetic peptide inhibitor of HIV-1 infection, binds to cell surface glycosphingolipids. Biochemistry.

[117] Yahi N., Fantini J., Baghdiguian S., Mabrouk K., Tamalet C., Rochat H., Van Rietschoten J., Sabatier J.-M. (1995). SPC3, a synthetic peptide derived from the V3 domain of human immunodeficiency virus type 1 (HIV-1) gp120, inhibits HIV-1 entry into CD4+ and CD4-cells by two distinct mechanisms. Proc. Natl. Acad. Sci. USA.

[118] Yahi N., Sabatier J.M., Baghdiguian S., Gonzalez-Scarano F., Fantini J. (1995). Synthetic multimeric peptides derived from the principal neutralization domain (V3 loop) of human immunodeficiency virus type 1 (HIV-1) gp120 bind to galactosylceramide and block HIV-1 infection in a human CD4-negative mucosal epithelial cell line. J. Virol..

[119] Faroux-Corlay B., Greiner J., Terreux R., Cabrol-Bass D., Aubertin A.-M., Vierling P., Fantini J. (2001). Amphiphilic anionic analogues of galactosylceramide: Synthesis, anti-HIV-1 activity, and gp120 binding. J. Med. Chem..

[120] Faroux-Corlay B., Clary L., Gadras C., Hammache D., Greiner J., Santaella C., Aubertin A.-M., Vierling P., Fantini J. (2000). Synthesis of single-and double-chain fluorocarbon and hydrocarbon galactosyl amphiphiles and their anti-HIV-1 activity. Carbohydr. Res..

[121] Kensinger R.D., Yowler B.C., Benesi A.J., Schengrund C.-L. (2004). Synthesis of novel, multivalent glycodendrimers as ligands for HIV-1 gp120. Bioconjugate Chem..

[122] Garg H., Francella N., Tony K.A., Augustine L.A., Barchi Jr J.J., Fantini J., Puri A., Mootoo D.R., Blumenthal R. (2008). Glycoside analogs of β-galactosylceramide, a novel class of small molecule antiviral agents that inhibit HIV-1 entry. Antivir. Res..

[123] Fantini J., Hammache D., Delézay O., Yahi N., André-Barrès C., Rico-Lattes I., Lattes A. (1997). Synthetic soluble analogs of galactosylceramide (GalCer) bind to the V3 domain of HIV-1 gp120 and inhibit HIV-1-induced fusion and entry. J. Biol. Chem..

[124] Gautret P., Lagier J.-C., Parola P., Meddeb L., Mailhe M., Doudier B., Courjon J., Giordanengo V., Vieira V.E., Dupont H.T. (2020). Hydroxychloroquine and azithromycin as a treatment of COVID-19: Results of an open-label non-randomized clinical trial. Int. J. Antimicrob. Agents.

[125] Fantini J., Di Scala C., Chahinian H., Yahi N. (2020). Structural and molecular modelling studies reveal a new mechanism of action of chloroquine and hydroxychloroquine against SARS-CoV-2 infection. Int. J. Antimicrob. Agents.

[126] Fantini J., Chahinian H., Yahi N. (2020). Synergistic antiviral effect of hydroxychloroquine and azithromycin in combination against SARS-CoV-2: What molecular dynamics studies of virus-host interactions reveal. Int. J. Antimicrob. Agents.

[127] Andreani J., Le Bideau M., Duflot I., Jardot P., Rolland C., Boxberger M., Wurtz N., Rolain J.-M., Colson P., La Scola B. (2020). In vitro testing of combined hydroxychloroquine and azithromycin on SARS-CoV-2 shows synergistic effect. Microb. Pathog..

[128] Jans D.A., Wagstaff K.M. (2020). Ivermectin as a broad-spectrum host-directed antiviral: The real deal?. Cells.

[129] Colson P., Raoult D. (2016). Fighting viruses with antibiotics: An overlooked path. Int. J. Antimicrob. Agents.

[130] Khoshnood S., Shirani M., Dalir A., Moradi M., Haddadi M.H., Sadeghifard N., Birjandi F.S., Yashmi I., Heidary M. (2022). Antiviral effects of azithromycin: A narrative review. Biomed. Pharmacother..

[131] Rizzo E. (2020). Ivermectin, antiviral properties and COVID-19: A possible new mechanism of action. Naunyn-Schmiedeberg’s Arch. Pharmacol..

[132] Rodrigo C., Fernando S.D., Rajapakse S. (2020). Clinical evidence for repurposing chloroquine and hydroxychloroquine as antiviral agents: A systematic review. Clin. Microbiol. Infect..

[133] Tan C.W., Sam I.-C., Chong W.L., Lee V.S., Chan Y.F. (2017). Polysulfonate suramin inhibits Zika virus infection. Antivir. Res..

[134] Gustafson K.R., Cardellina J.H., Fuller R.W., Weislow O.S., Kiser R.F., Snader K.M., Patterson G.M., Boyd M.R. (1989). AIDS-antiviral sulfolipids from cyanobacteria (blue-green algae). JNCI J. Natl. Cancer Inst..

[135] Xu M., Pradhan M., Gorshkov K., Petersen J.D., Shen M., Guo H., Zhu W., Klumpp-Thomas C., Michael S., Itkin M. (2022). A high throughput screening assay for inhibitors of SARS-CoV-2 pseudotyped particle entry. Slas Discov..

[136] Steffen I., Simmons G. (2016). Pseudotyping viral vectors with emerging virus envelope proteins. Curr. Gene Ther..

[137] Carter-Timofte M.E., Arulanandam R., Kurmasheva N., Fu K., Laroche G., Taha Z., van Der Horst D., Cassin L., van der Sluis R.M., Palermo E. (2021). Antiviral potential of the antimicrobial drug atovaquone against SARS-CoV-2 and emerging variants of concern. ACS Infect. Dis..

[138] Yan H., Sun J., Wang K., Wang H., Wu S., Bao L., He W., Wang D., Zhu A., Zhang T. (2021). Repurposing carrimycin as an antiviral agent against human coronaviruses, including the currently pandemic SARS-CoV-2. Acta Pharm. Sin. B.

[139] Du X., Zuo X., Meng F., Han C., Ouyang W., Han Y., Gu Y., Zhao X., Xu F., Qin F.X. (2021). Direct inhibitory effect on viral entry of influenza A and SARS-CoV-2 viruses by azithromycin. Cell Prolif..

[140] Ou T., Mou H., Zhang L., Ojha A., Choe H., Farzan M. (2021). Hydroxychloroquine-mediated inhibition of SARS-CoV-2 entry is attenuated by TMPRSS2. PLoS Pathog..

[141] Henß L., Beck S., Weidner T., Biedenkopf N., Sliva K., Weber C., Becker S., Schnierle B.S. (2016). Suramin is a potent inhibitor of Chikungunya and Ebola virus cell entry. Virol. J..

[142] Rojo J., Delgado R. (2004). Glycodendritic structures: Promising new antiviral drugs. J. Antimicrob. Chemother..

[143] Niaee M.S., Namdar P., Allami A., Zolghadr L., Javadi A., Karampour A., Varnaseri M., Bijani B., Cheraghi F., Naderi Y. (2021). Ivermectin as an adjunct treatment for hospitalized adult COVID-19 patients: A randomized multi-center clinical trial. Asian Pac. J. Trop. Med..

[144] Mahmud R., Rahman M.M., Alam I., Ahmed K.G.U., Kabir A.H., Sayeed S.J.B., Rassel M.A., Monayem F.B., Islam M.S., Islam M.M. (2021). Ivermectin in combination with doxycycline for treating COVID-19 symptoms: A randomized trial. J. Int. Med. Res..

[145] Seet R.C.S., Quek A.M.L., Ooi D.S.Q., Sengupta S., Lakshminarasappa S.R., Koo C.Y., So J.B.Y., Goh B.C., Loh K.S., Fisher D. (2021). Positive impact of oral hydroxychloroquine and povidone-iodine throat spray for COVID-19 prophylaxis: An open-label randomized trial. Int. J. Infect. Dis..

[146] Li X., Wang Y., Agostinis P., Rabson A., Melino G., Carafoli E., Shi Y., Sun E. (2020). Is hydroxychloroquine beneficial for COVID-19 patients?. Cell Death Dis..

